# *De novo* transcriptome assembly of cold stressed clones of the hexaploid *Sequoia sempervirens* (D. Don) Endl.

**DOI:** 10.1038/s41597-020-00576-1

**Published:** 2020-07-17

**Authors:** Natalie Breidenbach, Vadim V. Sharov, Oliver Gailing, Konstantin V. Krutovsky

**Affiliations:** 1 Department of Forest Genetics and Forest Tree Breeding, Georg-August University of Göttingen, 37077 Göttingen, Germany; 2 Laboratory of Genomic Research and Biotechnology, Federal Research Center “Krasnoyarsk Science Center of the Siberian Branch of the Russian Academy of Sciences”, 660036 Krasnoyarsk, Russian Federation; 3 Department of High Performance Computing, Institute of Space and Information Technologies, Siberian Federal University, 660074 Krasnoyarsk, Russian Federation; 4 Center for Integrated Breeding Research, George-August University of Göttingen, 37075 Göttingen, Germany; 5 Laboratory of Forest Genomics, Genome Research and Education Center, Institute of Fundamental Biology and Biotechnology, Siberian Federal University, 660036 Krasnoyarsk, Russian Federation; 6 Laboratory of Population Genetics, N. I. Vavilov Institute of General Genetics, Russian Academy of Sciences, 119333 Moscow, Russian Federation; 7 Department of Ecosystem Science and Management, Texas A&M University, College Station, TX 77843-2138 USA

**Keywords:** Gene expression, Abiotic

## Abstract

Coast redwood is a very important endemic conifer timber species in Southern Oregon and Northern California in the USA. Due to its good wood properties and fast growth rate it can be considered as a prospective timber species also in other countries with similar or changing toward similar climatic conditions due to global climate warming, such as Germany. In general, it is frost sensitive and suffers from freezing temperatures. To study genetic mechanisms of frost resistance in this species and to select the most frost tolerant trees we tested 17 clones in climate control chamber experiments and generated two *de novo* assemblies of the coast redwood transcriptome from a pooled RNA sample using Trinity and CLC Genomic Workbench software, respectively. The hexaploid nature of the coast redwood genome makes it very challenging to successfully assemble and annotate the coast redwood transcriptome. The *de novo* transcriptome assembly generated by Trinity and CLC considering only reads with a minimum length of 180 bp and contigs no less than 200 bp long resulted in 634,772 and 788,464 unigenes (unique contigs), respectively.

## Abstract (summary)

**Table:** 

Measurement(s)	RNA • transcriptome • sequence_assembly • sequence feature annotation
Technology Type(s)	RNA sequencing • sequence assembly process • sequence annotation
Factor Type(s)	temperature treatment
Sample Characteristic - Organism	Sequoia sempervirens

Machine-accessible metadata file describing the reported data: 10.6084/m9.figshare.12554549

## Background & Summary

Coast redwood (*Sequoia sempervirens* (D. Don) Endl.) is an endemic forest tree conifer species occupying a narrow range along the Pacific Northwest coast in southern Oregon and northern California, USA. It is a valuable timber species characterized by fast growth rate and good quality wood^1^. The species has been planted successfully in some other countries for commercial wood production^2^, but in Germany it is used currently rather as a decorative, exotic species, mainly due to its sensitivity to freezing temperatures. However, some coast redwood trees survived freezing temperatures in Germany demonstrating cold-tolerance. Considering global warming and climate change, this species can be potentially considered as a prospective commercial timber species for future German sustainable forestry. To study genetic mechanisms of cold-resistance and to select frost-resistant coast redwood trees we tested replicates of 17 different coast redwood clones of diverse origin (Table 1) in a climate control chamber under a freezing temperature of up to −10 °C. Samples included the ‘Filoli-phenotype’ clones and clones from two trees growing in Germany that are considered as frost resistant. RNA isolated from 12 clones from different temperature treatments was used to generate two *de novo* assemblies of a coast redwood transcriptome using Trinity and CLC Genomic Workbench software considering only reads with a minimum length of 180 bp and contigs no less than 200 bp long. Coast redwood is a hexaploid species and is very difficult to study. Its genome has only recently been sequenced, and the genome assembly has been made publicly available (https://nealelab.ucdavis.edu/redwood-genome-project-rgp, accessed in May 2019), but it is still unpublished and not annotated. Published transcriptome data are also limited^3^. Two transcriptome assemblies obtained in our study provide additional invaluable genomic resources and can support further coast redwood genetic studies including those concerning response of this and other conifer species to frost stress or other environmental stresses in general. We also hope that our experience with *de novo* sequencing, assembling and annotating the transcriptome of this difficult non-model polyploid species can help other similar studies.

**Table 1:** Coast redwood clones tested in climate control chamber at freezing temperature −10 °C.

Clone	Origin	Latitude	Longitude
ANG3*	Angwin, USA	38.534967	−122.429347
ANG4*	Angwin, USA	38.534967	−122.429347
B167	Freshwater Creek, USA	40.75	−124.05
BLU71*	Filoli Phenotype, USA	unknown	unknown
BLU94*	Filoli Phenotype, USA	unknown	unknown
L19	Patrick Creek, USA	41.816667	−123.933333
L20	Patrick Creek, USA	41.816667	−123.933333
NAV1*	Navarro, USA	39.151944	−123.541944
NAV3	Navarro, USA	39.151944	−123.541944
NO1*	Northern California, USA	unknown	unknown
NO3*	Northern California, USA	unknown	unknown
SA1*	Santa Cruz, USA	36.971944	−122.026389
SA2	Santa Cruz, USA	36.971944	−122.026389
SF1*	Sequoiafarm Kaldenkirchen, Germany	51.308117	6.171964
SF3*	Sequoiafarm Kaldenkirchen, Germany	51.308117	6.171964
WI3	Winchuk, USA	42.05	−124.215278
WI4*	Winchuk, USA	42.05	−124.215278

*These 11 clones with successful RNA extraction were included in the pooled sample used for the sequencing and *de novo* transcriptome assembly.

## Methods

### Plant material

**Fig. 1:**
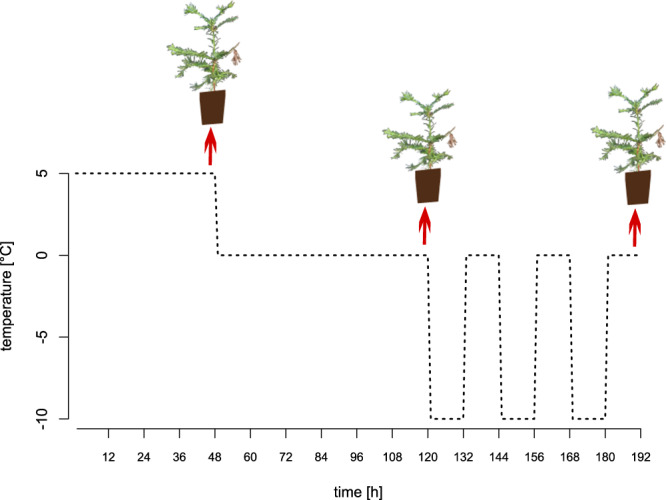
Temperature level and duration during frost experiment in a climate control chamber. Arrows indicate harvest of coast redwood plants after each temperature treatment at 5 °C, 0 °C, and −10 °C.

In January 2018, 17 nine-month-old clones were tested in a climate chamber under controlled light and temperature conditions (Fig. 1). The frost experiment followed a modified version of the experimental design used by Arbaoui *et al*.^4^ and consisted of a hardening phase at 5 °C for 48 h and at 0 °C for 72 h with 12 h of dark and a low light intensity for 12 h followed by freezing temperatures at −10 °C for 12 h simulating a freezing winter night and 12 h at 0 °C with lights on simulating a winter day, respectively, repeated twice. The experiment started with lights off. For each temperature treatment at 5 °C, 0 °C, and −10 °C, the positions of 2–4 ramets per clone were randomly rearranged within the climate control chamber to minimize possible effects of micro-spatial climatic differences in the chamber (Fig. 1). After each treatment a single entire ramet of each clone was harvested and immediately frozen in liquid nitrogen. The samples were stored at −60 °C until RNA extraction.

### RNA extraction

Needle tissue was ground in liquid nitrogen, and its RNA was extracted following the CTAB protocol of Chang *et al*.^5^ After extraction, each sample was treated with 1 µl DNAse (Thermo Fisher, Waltham, MA, USA). RNA quality and integrity were assessed using the Fragment Analyzer System and standard sensitivity RNA Analysis Kit DNF-471 (Agilent Technologies, Inc., Santa Clara, CA, USA). All samples selected for sequencing had an RNA integrity number over 8.

### RNA sequencing

**Fig. 2:**
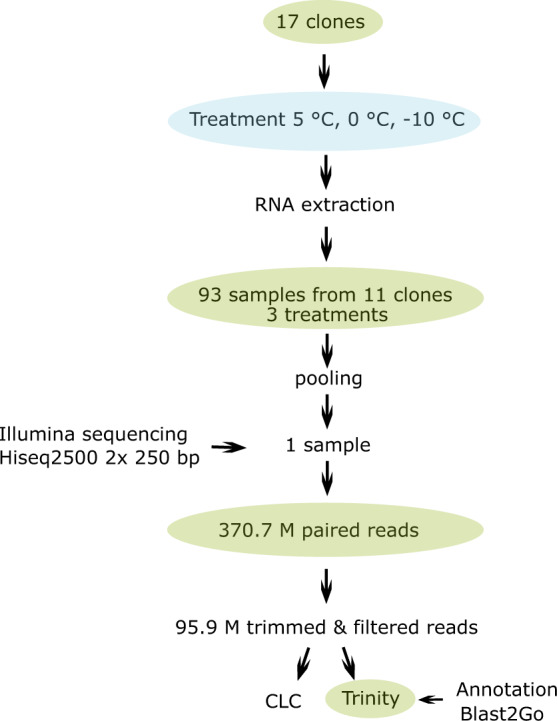
Overview of the experiment and analysis. 17 coast redwood clones were tested at three temperature levels. RNA was isolated in sufficient quality and quantity for sequencing from 93 samples representing 11 different clones and equimolarily pooled. In total, ~370.7 M paired-end reads were generated. *De novo* assembly was done with Trinity and CLC Genomic Workbench software using ~95.9 M paired-end quality trimmed and filtered by minimum size of 180 bp reads, respectively. The Trinity *de novo* assembly based on the filtered reads was annotated using Blast2GO Pro.

Only clones with sufficient quality RNA at all three temperature treatments were used for sequencing. In total, ramets of 11 clones representing all three temperature treatments resulted in 93 samples that were equimolarily pooled into one sample and sequenced at the NGS Integrative Genomics Core Unit, University Medical Center, Göttingen (Fig. 2). A paired-end (PE) RNA-seq library was prepared using the pooled RNA sample and a non-stranded, massively-parallel cDNA sequencing (mRNA-Seq) protocol with the TruSeq mRNA prep Kit (Cat. No. RS-122-2101) from Illumina, Inc. (San Diego, CA, USA). The ligation step in the protocol was optimized by diluting the adapter concentrations to increase ligation efficiency (>94%), and the number of PCR cycles was reduced to avoid PCR duplication artefacts as well as primer dimers in the final library. The fluorometric based QuantiFluor™dsDNA System (Promega GmbH, Mannheim, Germany) was used for accurate quantitation of the cDNA library. The size of the final cDNA library was determined by using the dsDNA 905 Reagent Kit (Agilent Technologies, Inc., Santa Clara, CA, USA) with sizing range of 35–500 bp and resolution of 3–5 bp at 300 bp on average. The PE library was sequenced in two flow cell lanes on the Illumina HiSeq 2500 with a rapid mode and 2 × 250 cycles. Sequence images were translated to BCL files by the Illumina software BaseCaller and then demultiplexed to fastq files using bcl2fastq v2.17.1.14 software. In total, ~370.7 M paired-end reads were generated, and after the quality trimming, minimum length filtering at 180 bp, and contamination removing ~95.9 M paired-end reads with an average length of 424 bp and total ~40.7 Gb were used for transcriptome assembly and submitted to the NCBI Genbank SRA public database^6^.

#### Transcriptome *de novo* assembly

The reads were trimmed using Phred quality score 30 and base call accuracy of 99.9%. As a result, 1.35% of the reads were trimmed, which reduced the average read length by about 0.5 bp. Then, only reads with a minimum length of 180 bp were used for transcriptome assembly. Based on these reads two transcriptome *de novo* assemblies were generated using two programs - Trinity and CLC Genomics Workbench, respectively. The Trinity assembly was normalized to a maximum read coverage of 30X. This value is less than the Trinity default of 50X, but it is in agreement with a value of at least 30, which is recommended by the authors of this algorithm in the supplement (S4) to Haas *et al*.^7^ The CLC assembly was carried out with default settings considering a minimum unigene (contig) length of 200 bp. Assemblies from both softwares showed signs of inflation and overrepresentation (Table 2). The best results were obtained for the transcriptome assembled using the Trinity software. This assembly based on the filtered reads was annotated using Blast2GO Pro. MIcroSAtellite (MISA) identification online tool^8^ was used with default parameters to identify microsatellite loci (Summary of the microsatellite loci identified by the MISA tool in the Trinity assembly based on the filtered reads^9^) with di-, tri-, tetra-, penta- and hexanucleotide motifs in this Trinity assembly, and PCR primers (PCR primers designed for the microsatellite loci identified by the MISA tool in the Trinity assembly based on the filtered reads using Primer3 online tool^9^) were designed for these loci using the Primer3 tool.

**Table 2:** Summary statistics of two coast redwood transcriptome *de novo* assemblies generated using Trinity and CLC Genomics Workbench software considering only reads with a minimum length of 180 bp and contigs no less than 200 bp long, respectively.

Unigenes/contigs	Trinity	CLC
Total number	622955	773507
L50	89696	206876
Max length, bp	29218	21583
N50, bp	1391	419
N80, bp	457	240
Total length, Mbp	522.0	306.1

#### Transcriptome functional annotation

Using *blastx* search with *gilist* taxid option for “Green plants” homologs were identified for the contigs of the Trinity assembly in the GenBank *nr* database. Then, the *blastx* output data were sorted out by the Blast2GO PRO program using the “Gene Ontology Mapping” function.

## Data Records

The filtered and cleaned original RNA sequencing data have been deposited at the NCBI Sequence Read Archive under the SRA study accession SRP227297 (https://identifiers.org/ncbi/insdc.sra:SRP227297). The contigs for the Trinity^10^ and CLC^11^ transcriptome assemblies have been deposited as Transcriptome Shotgun Assembly (TSA) projects at DDBJ/EMBL/NCBI GenBank under the accession numbers GIBU00000000 (https://identifiers.org/ncbi/insdc:GIBU00000000) and GIDF00000000 (https://identifiers.org/ncbi/insdc:GIDF00000000), respectively. Functional annotation of the Trinity transcriptome assembly is available as a supplementary gff file at figshare (Functional annotation of the Trinity transcriptome assembly^9^). Summary of the microsatellite loci identified by the MISA tool in the Trinity assembly based on the filtered reads is available as a supplementary excel file at figshare (Summary of the microsatellite loci identified by the MISA tool in the Trinity assembly based on the filtered reads^9^). PCR primers designed for these microsatellite loci using Primer3 online tool are available as a supplementary excel file at figshare (PCR primers designed for the microsatellite loci identified by the MISA tool in the Trinity assembly based on the filtered reads using Primer3 online tool^9^).

## Technical Validation

### Quality control

**Fig. 3:**
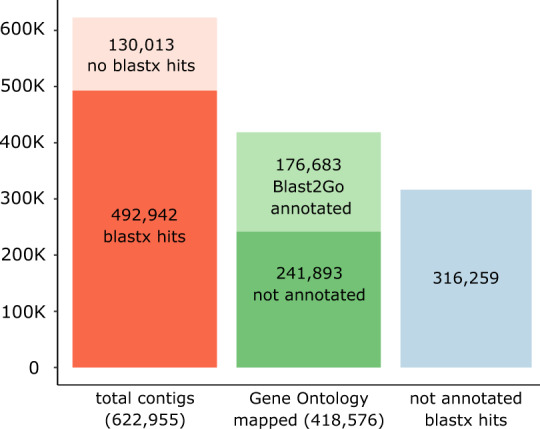
Annotation results for the Trinity assembly based on the filtered reads.

**Fig. 4:**
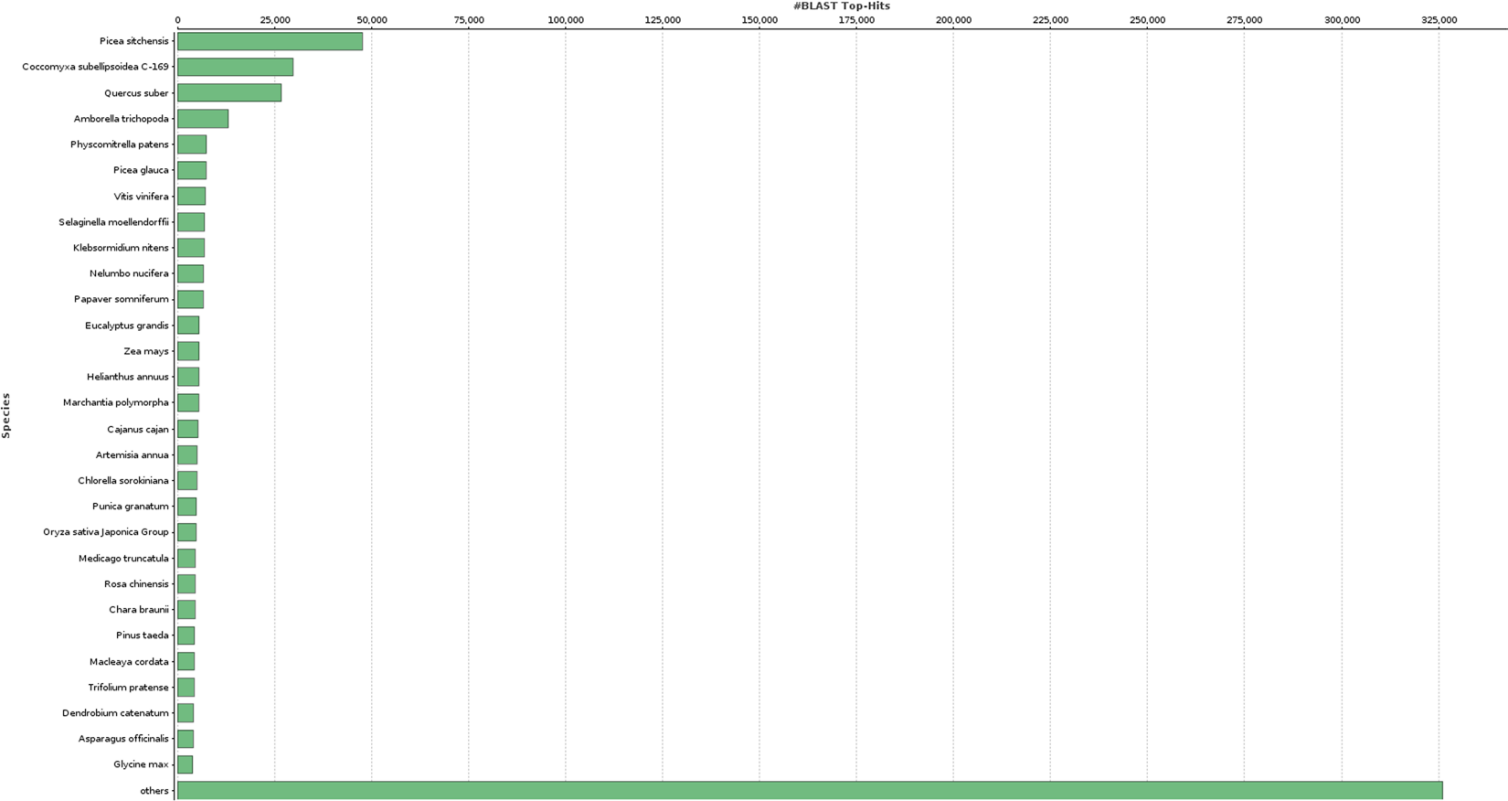
Number of top-hit coast redwood sequences matching other species sequences based on blastx.

The quality check was done using FastQC^12^ v. 0.11.5. Using *blastx* and Blast2GO, 418,576 (67%) out of total 622,955 unigenes (contigs) were mapped and 176,683 (28%) annotated; 130,013 (21%) had no blast hits, and 316,259 (51%) had hits but were not annotated (Fig. 3). The largest number of blast hits represented *Picea sitchensis*, followed by the algae *Coccomyxa subellipsoidea* C-169 and *Quercus suber* (Fig. 4).

### Gene Ontology analysis

**Fig. 5:**
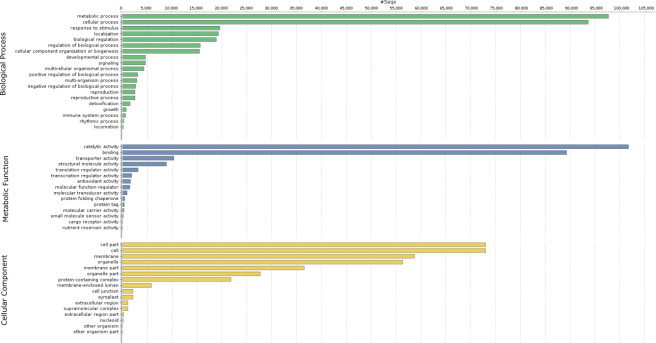
The Gene Ontology (GO) term distributions for biological processes (green), metabolic function (blue) and cellular components (yellow).

**Fig. 6:**
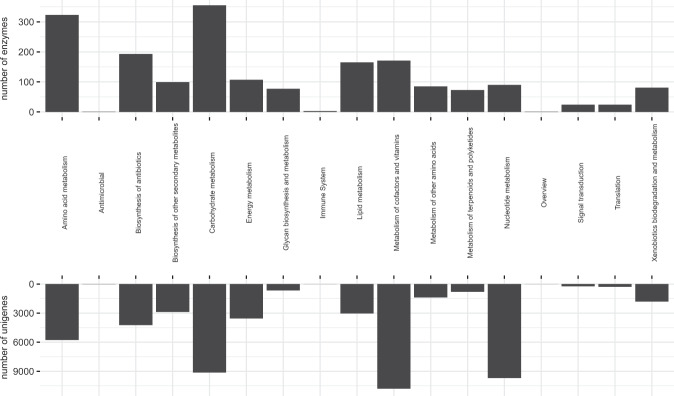
Number of enzymes (upper chart) and unigenes (lower chart) partitioned into 17 classes according to the KEGG (Kyoto Encyclopedia of Genes and Genomes) biological pathways.

Distribution of the Gene Ontology (GO, http://geneontology.org) terms demonstrated that within the biological processes the most frequent were metabolic and cellular processes that were represented by more than 90 000 unigene (contig) sequences. Response to stimulus was the third most common process represented by approximately 20 000 unigene (contig) sequences (Fig. 5). More than 100 000 unigene (contig) sequences were associated with catalytic activity in the metabolic functions. Unigene (contig) sequences associated with cell parts and cell membrane were the most common in the cellular components level. The KEGG (Kyoto Encyclopedia of Genes and Genomes; https://www.kegg.jp) annotation revealed that annotated sequences represented mostly carbohydrate, amino acid, cofactor, and vitamin related metabolism (Fig. 6). Many of them could be potentially involved in response to frost.

### BUSCO analysis

To test transcriptome assemblies for completeness, a search for conserved orthologous genes was done in both transcriptome assemblies using the Benchmarking Universal Single-Copy Orthologs (BUSCO) program^13^. The plant databases viridiplantae_odb10 (“green plants”, creation date: 2019–11–20, number of species: 57, number of BUSCOs: 425) and embryophyta_odb10 (“land plants”, creation date: 2019–11–20, number of species: 50, number of BUSCOs: 1614) were used as lineage-specific datasets. The results are presented in Table 3 and demonstrate that both assemblies are rather complete, but the Trinity assembly is almost twice as complete as the CLC one and includes 1409 (87.3%) or 397 (93.4%) of complete BUSCOs depending on the Embryophyta or Viridiplantae dataset, respectively.

**Table 3:** Summary statistics of the BUSCO analysis of two coast redwood transcriptome *de novo* assemblies generated using Trinity and CLC Genomics Workbench software.

Transcriptome assembly	Trinity	CLC
**Viridiplantae dataset**		
Complete BUSCOs	397 (93.4%)	205 (48.2%)
Complete and single copy BUSCOs	136 (32.0%)	176 (41.4%)
Complete and duplicated BUSCOs	261 (61.4%)	29 (6.8%)
Fragmented BUSCOs	25 (5.9%)	186 (43.8%)
Missing BUSCOs	3 (0.7%)	34 (8.0%)
Total BUSCO groups searched	425	425
**Embryophyta dataset**		
Complete BUSCOs	1409 (87.3%)	570 (35.3%)
Complete and single copy BUSCOs	517 (32.0%)	509 (31.5%)
Complete and duplicated BUSCOs	892 (55.3%)	61 (3.8%)
Fragmented BUSCOs	87 (5.4%)	491 (30.4%)
Missing BUSCOs	118 (7.3%)	553 (34.3%)
Total BUSCO groups searched	1614	1614

### Mapping transcripts to the reference coast redwood genome assembly

Transcripts from both transcriptome assemblies were mapped to the reference coast redwood genome assembly (NCBI Assembly accession number GCA_007258455.1) using magic-blast (https://ncbi.github.io/magicblast/). In total, 96.9% and 98.5% of transcripts in the CLC and Trinity assemblies, respectively, were mapped to the genome. It is worth noting that we tried also a few other programs such as STAR, HISAT2, exonerate, and nucmer, but they could not handle mapping the large transcriptome assemblies to the large genome, and some of these programs (for example STAR) were designed to map short reads rather than relatively long transcripts. It is hard to predict how many genes can be expected in a coast redwood genome considering its hexaploid nature. The coast redwood draft genome assembly is neither annotated nor published yet. There are also only a few conifer species (all diploid) with annotated genomes (see Table 3 in Mosca *et al*.^14^ for review). Based on these data it ranges from 47,602 in *Pinus taeda* to 102,915 in *Picea glauca*. Therefore, we can easily expect as many as 600,000 genes. However, we have to emphasize that the presented assemblies are raw *de novo* ones and are likely highly redundant.

### Microsatellite discovery and testing

The MISA search of the 622,955 unigene (contig) sequences found 37,164 microsatellite loci in 31,968 sequences. Among them, 19,048 SSRs represented microsatellite loci with mononucleotide motifs, 9,795 - dinucleotide, 7,346 - trinucleotide, 669 - tetranucleotide, 132 - pentanucleotide, and 174 - hexanucleotide motifs (Summary of the microsatellite loci identified by the MISA tool in the Trinity assembly based on the filtered reads^9^). Using the online software Primer3 PCR primer pairs were successfully designed for 28,285 microsatellites: 14,806 with mononucleotide motifs, 6,226 - dinucleotide, 5,601 - trinucleotide, 432 - tetranucleotide, 77 - pentanucleotide, 95 – hexanucleotide, and 1,048 compound or complex motifs (PCR primers designed for the microsatellite loci identified by the MISA tool in the Trinity assembly based on the filtered reads using Primer3 online tool^9^). Twenty PCR primer pairs with unique single-copy annealing sites in both Trinity transcriptome and reference genome assemblies and with similar melting temperatures for multiplexing were selected, and respective oligos were synthesized with forward primers containing the M13 tail (5′-CACGACGTTGTAAACGAC-3′) and reverse primers containing the pig-tail (5′-GTTTCTT-3′). The M13 primer were labelled either by 6-FAM or HEX (Sigma Aldrich Inc., St. Louis, MO). The same touch-down PCR program was used for all 20 PCR primer pairs following the protocol described in Breidenbach *et al*.^15^ The PCR products were separated and visualized using the ABI Genetic Analyser 3130xl with GENSCAN ROX 500 as an internal size standard. The primers were tested in a population sample of eight trees. Their DNA was isolated from needles or cambium using the DNeasy Plant Kit (Qiagen, Hilden, Germany) following the manufacturer’s instructions. The isolated DNA was diluted in ddH_2_O 1:10 for PCR amplification and stored at −20 °C. All primer pairs amplified alleles of expected size, and 14 markers were polymorphic (20 PCR primer pairs tested^9^) and can be used in different applications.

## Data Availability

Blast2GO PRO: https://www.blast2go.com/blast2go-pro BUSCO v4.0.5: https://busco.ezlab.org FastQC v0.11.5: https://www.bioinformatics.babraham.ac.uk/projects/fastqc Magic-BLAST v1.5.0: https://ncbi.github.io/magicblast MISA: http://pgrc.ipk-gatersleben.de/misa/misa.html PRIMER3: https://github.com/primer3-org/primer3 Trimmomatic v.0.35: http://www.usadellab.org/cms/?page=trimmomatic Trinity v2.8.4: https://github.com/trinityrnaseq/trinityrnaseq/wiki
